# Facilitators and barriers influencing utilization of services provided by community midwives in district Thatta, Pakistan: a qualitative exploratory study

**DOI:** 10.1186/s12884-022-04823-8

**Published:** 2022-06-22

**Authors:** Bakhtawar M. Hanif Khowaja, Anam Shahil Feroz, Sarah Saleem

**Affiliations:** 1 Department of Obstetrics and Gynecology, The Aga Khan University, Stadium Road, PO Box 350, Karachi, 74800 Pakistan; 2 Department of Community Health Sciences, The Aga Khan University, Stadium Road, PO Box 3500, Karachi, 74800 Pakistan

**Keywords:** Community midwives, Community midwives services, District Thatta, Pakistan, Facilitators and barriers

## Abstract

**Background:**

To address the issue of high maternal mortality, the Government of Pakistan initiated a community midwifery program in 2006 to provide skilled birth attendance to women living in rural areas. Despite a large investment in the community midwifery program, research evidence from rural districts of Pakistan suggests that the utilization of maternal and newborn services through community midwives is very low. This exploratory study aimed to understand the facilitators and barriers influencing community midwives’ services utilization in district Thatta.

**Methods:**

A qualitative study was conducted in the rural district Thatta, Pakistan. Key-informant interviews (KIIs) were conducted with district officials of the Health department (Thatta), Maternal and Newborn Child Health Program, and Midwifery Association of Pakistan (MAP). In-depth Interviews (IDIs) were conducted with midwifery students who were currently enrolled in the midwifery program of the district; trained community midwives providing services in district Thatta, and trained community midwives not continuing their profession. IDIs were also conducted with community women to explore their views about the scope of midwifery practice and the factors influencing the utilization of community midwives’ services in district Thatta, Pakistan. Data were analyzed using qualitative thematic analysis.

**Results:**

A total of 25 interviews (KIIs = 5; IDIs = 20) were conducted. Two overarching themes were identified: (I) community midwives’ skills and competencies; and (II) ownership and supportive supervision. The major hindering factors for community midwives’ service utilization included deficiencies in community midwives’ training particularly in clinical hands-on training, lack of ownership of community midwifery program, and lack of service structure by the CMWs regulatory body.

**Conclusion:**

The study has identified serious gaps in the CMWs program at the level of training and supervision of midwives in Pakistan. The study has also identified factors related to the training of CMWs that could facilitate the program in the context of Pakistan and similar settings.

**Supplementary Information:**

The online version contains supplementary material available at 10.1186/s12884-022-04823-8.

## Background

It is estimated globally that every day about 830 women die due to childbirth and pregnancy related causes, which are preventable [1]. Access to antenatal, delivery, and postnatal care is crucial in decreasing maternal and newborn deaths [1]. The proportions of women who do not receive antenatal and perinatal services are susceptible to complications and many of them face medical interventions that increase financial as well as physical health burdens [2]. These complications can be prevented by services provided by skilled health workers including a trained doctor, nurse, or midwife [3]. World Health Organization emphasizes skilled care at birth to reduce maternal and newborn deaths [4].

There is collective evidence about the role of midwives in positive pregnancy outcomes for women and newborns [5]. Midwifery is an ancient practice from the time of early Egyptian and Roman times and has gained global attention today [6]. The number of maternal and newborn mortality has significantly decreased with the integration of midwifery-led models worldwide [7]. Many countries such as Sri Lanka, Thailand, and Malaysia succeeded in improving maternal morbidities and mortalities by increasing investment in training and deployment of midwives, giving high credibility to the profession of midwifery [8]. Sri Lanka reduced its maternal mortality rate (MMR) from 2000 to 31/100 000 live births between 1930 and 2011. Thailand and Malaysia reduced their MMRs from 425 and 275 in the year 1960 to less than 30/100 000 live births in 2010 respectively using the same strategy [8]. The number of maternal and newborn mortality remains high in Asia and sub-Saharan Africa, where less than 50% of all births are assisted by a skilled birth attendant [9].

In Pakistan, complications during pregnancy and childbirth are the prominent causes of death in women aged 15–45 years, accounting for 12% of all deaths of women of child-bearing age [10]. The MMR in Pakistan recorded 186 deaths/100,000 live births in 2020 [11]. The total NMR in Pakistan has been reported at 44 deaths per 1000 live births in 2015 [11]. However, in rural areas, the NMR has been reported much higher at 62 deaths per 1000 live births [11]. National figures show that 62 percent of the population resides in rural areas with poor healthcare infrastructure and limited basic facilities related to maternal and newborn health services [12]. The factors for poor maternal and neonatal outcomes include poor utilization of ANC services, with only 52.8% of Pakistani pregnant women attending three or more ANC visits [12].

Drawing on this evidence, Pakistan introduced a community health worker-based program in 2006, named, the Maternal, Newborn and Child Health (MNCH) program in an attempt to improve maternal and newborn health indicators [13]. There are a number of vertical programs under the MNCH program such as Lady Health Worker (LHW), Lady Health Visitor (LHV), Family Planning (FP), Expanded Program on Immunization (EPI), and, CMW program. The CMW program offers training and deployment of community midwives to increase skilled birth attendance in rural communities. [14].

The government of Pakistan underwent a major reform of devolution in 2011, resulting in reorganizing the health system at the federal, provincial, and district levels [15]. Midwifery schools have been organized in all provinces [16]. Presently, there are more than 100 midwifery schools in Pakistan and are divided between public and private institutions [17].

The community midwifery training originated as a 12 months training program in 2006 with an advancement to 24 months training period in 2018 [18]. CMWs education program has been grounded on the Midwifery Model of Care and adapted from International Confederation of Midwives (ICM) competencies to provide knowledge and skills for maternal and newborn health services [19]. Midwifery education in the country is based on the traditional model and comprises two major components of classroom teaching and hands-on practical training [20]. After successful completion of the training, the community midwife is expected to serve in the community she has been selected from to establish home-based clinics to provide ANC, childbirth, and PNC services to rural women [21]. The Pakistan Nursing Council (PNC) is the accrediting body and provides licenses to all midwifery practitioners [18].

Midwives are usually the first point of care for women during their reproductive health period in rural settings [13]. According to the 2010 UNICEF annual report, most pregnant women in Pakistan go to district hospitals instead of seeking care in community centers because these facilities lack trained midwives, medicine, equipment, and transportation in case of emergencies [22].

National figures show that more than 7000 community midwives are trained each year however there is an absence of data on CMWs deployment [23]. Approximately 40% of the deliveries took place at home or outside a healthcare facility and 52% of deliveries were performed by non-skilled healthcare personnel [11]. This puts both mothers and newborns at risk of health complications [12]. A cross-sectional study conducted in 10 selected districts of Sindh showed that around 22.5% of the deliveries were conducted by midwives employed at public health facilities and only 6% of the deliveries were performed by CMWs working at their birthing stations [24].

Despite a large investment by the government of Pakistan in the CMW program, there is poor CMW program functioning in rural districts of Pakistan [25]. The weak policy elements such as ownership of the CMW program, unsteady governance of the health system, inadequate health sector financing, and management of human resources cause an overwhelming challenge to sustainability and utilization of community midwives’ services [25].

There is a need to better understand the significance and scope of community midwives for the provision of maternal and newborn health services in rural districts of Pakistan and to explore factors influencing CMWs’ utilization of services. To do so, we focused on the views of community midwives and community women of district Thatta; and health officials involved in the training and implementation of the CMW program.

## Methods

### Study setting

In view of the lack of data on poor CMW program functioning in rural areas of province Sindh, the study site selected was rural district Thatta of Province Sindh. Thatta is a predominantly rural district bordering Karachi and Hyderabad, the two largest cities in the province of Sindh with a total population of 979,817 [26]. Despite being adjacent to these urban centers, Thatta was ranked 90th of 114 districts in Pakistan on the Human Development Index in 2017 and was in the bottom five among the 24 districts within the province of Sindh [26]. Reports from 2016 show that the proportion of women delivering at healthcare facilities in Thatta is higher than the national average [11].

There is a midwifery school in district Thatta that offers 24 months diploma program and trains more than 30 CMWs each year [27]. The current district data displays that out of 110 deployed and registered CMWs, only 17 CMWs report to the MNCH program monthly. The MNCH program in district Thatta is funded and managed by the provincial Government of Sindh [27], and hence resource constraints of the provincial government are reflective of the situation in most of the districts of the province Sindh. This made the area an ideal location for exploring the challenges faced by community midwives.

### Design and study team

**Fig. 1:**
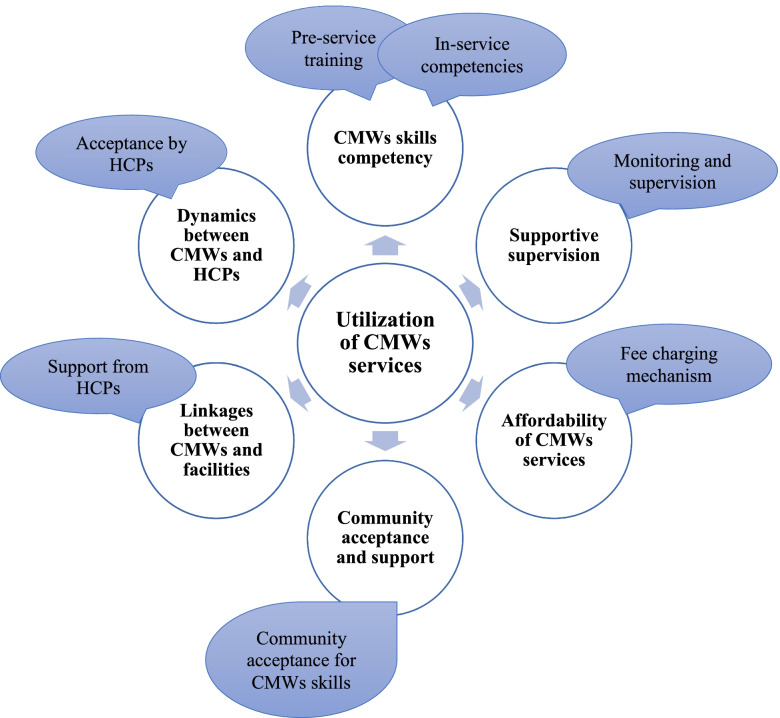
The six factors influencing CMWs services utilization

In order to understand the factors that influence community midwives’ services utilization in district Thatta, the study adapted the community midwifery model, guided by Chitral Child Support Program (CCSP) strategy [28]. The CCSP project was carried out in North Chitral implementing the community midwifery model to enhance the utilization of CMWs services [28]. CMWs were trained and deployed in their communities with necessary equipment and medicines, and a system for supportive supervision [28]. The project achieved significant improvements in access to care from skilled and trained CMWs. The CMW model (Fig. 1) is comprised of six major factors: (I) CMWs skills competencies, (II) supportive supervision, (III) affordability for CMWs services, (IV) community acceptance and support, (V) linkages between CMWs and facilities, and (VI) dynamics between CMWs and other health care providers.

We explored two factors from the CMW model (I) CMWs’ skills competencies, and (II) supportive supervision in the context of rural Thatta to explore the supportive actions provided to CMWs for their training, deployment, and implementation of the services. These factors guided our research methods in terms of structuring the data collection methods, analysis, and reporting of findings. The result of our study will inform recommendations for training and supportive supervision of the CMW program in rural areas of Pakistan.

We used an exploratory qualitative study design by conducting key-informant interviews (KIIs) and in-depth interviews (IDIs) and using a semi-structured interview guide. Three researchers from Aga Khan University designed and conducted the study for the master’s thesis for Health Policy and Management Degree requirement by the first author. All researchers had expertise in relevant areas: SS and AF (experience in health policy and the design and implementation of qualitative research and data analysis); BK (master’s student with public health and health policy background). The standards for reporting qualitative research guidelines were used (see Additional file 1).

### Study participants

KIIs were conducted with officials from the District health department who were involved in CMWs training and deployment; these were the District Health Officer, District coordinator of the MNCH program, Principal of the midwifery school of district Thatta, General Secretary of Midwifery Association of Pakistan, and Director of General nursing and midwifery program. In-depth interviews were conducted with three categories of CMWs including midwifery students who were currently enrolled in the midwifery school of Thatta; trained community midwives providing services in district Thatta; trained community midwives who have left the midwifery profession; and community married women from district Thatta. The aim of KIIs and IDIs was to explore and understand the current scope of midwives in the district and to explore the facilitators and barriers in CMWs’ training and supportive supervision that influence the utilization of their services.

### Data collection and reflexivity

Semi-structured data collection tools were developed in the English language and translated into local languages Urdu and Sindhi to capture the perceptions of the study participants. The interviews were conducted in Urdu and Sindhi languages between August 2020 and December 2020. The interview guides were developed with the aim of understanding and exploring factors influencing the utilization of CMWs services in rural areas. We included key themes with several specific questions: (1) CMWs’ skills competencies, such as their classroom training, clinical training, skills development and competencies, and their admission criterion (2) Supportive supervision, such as the support from governments and the MNCH program, support from PNC and training school, supervision of training schools, birthing stations, and their services.

The interview guides were tailored for each category of participants. The research team reviewed the interview guide for content and flow and trialed the guide for the length of time and appropriateness of the questions. The detailed interview guides are provided as Additional file 2.

Due to the restrictions in the district because of the Covid-19 situation, all the interviews were conducted via telephonic calls. These interviews were scheduled according to participants’ preference of time and were audio-recorded following verbal informed consent from study participants. Each interview lasted between 45 to 60 min. The KIIs and IDIs were carried out until the data became saturated and no new information emerged. The lead author was fluent in local languages and carried out all the interviews.

### Ethical approval

The Ethics Review Committee of Aga Khan University, Karachi, Pakistan, gave the ethical approval for the study (2020–3391-11,138). Informed verbal consent was translated into Urdu and Sindhi language. Every aspect of the consent form was well explained to study participants including the purpose of the study, possible risks, and discomforts, possible benefits, the confidentiality of information, and data storage for 7 years in a password-protected place with the research team. Informed consent included permission to audio record the interviews and use of anonymized quotes.

### Data analysis

Data were collected and analyzed through an iterative process and data collection was ceased once saturation was achieved. The audio recordings were transcribed into Sindhi and Urdu languages and later translated into English language and cross-examined by all the researchers of the study team. Two themes from the CMW model were used to guide data coding, data analysis, and reporting of the findings. A qualitative thematic analysis approach was adopted and codes were formulated deductively from the transcripts for the predefined themes [29]. A codebook was developed based on the two main themes. Additional codes that emerged from the transcripts were added where possible. The data analysis was done manually. Three researchers (SS, AF, BK) participated in refining the codebook to reach a final consensus and resolve any discrepancies [30]. To adapt two themes of the CMW model to our study, we removed those codes that were not reflected in the transcript. In detail, we described CMW’s skills and competencies and Supportive supervision (supplemental file- thematic map). Study rigor was ensured by triangulation of different data sources (health officials, midwifery students, trained community midwives, and community women) and data collection methods (KIIs and IDIs).

## Results

A total of 25 semi-structured interviews were conducted. Five KIIs were conducted with health officials who were involved in community midwives’ training and deployment (*n* = 5). Table 1 describes the characteristics of health officials. IDIs were conducted with five participants from each category of midwives such as midwifery students (*n* = 5) (see Table 2), community midwives working in the district (*n* = 5), community midwives trained but not working in their professions (*n* = 5) (see Table 3), and community women (*n* = 5) (see Table 4).

**Table 1:** Characteristics of Health Officials

Health officials	*N* = 5
**Association with CMW program**	
Less than 5 years	2
5 to 10 years	2
More than 10 years	1
**Location/supervision at:**	
District level	3
Provincial level	1
Federal level	1
**Role in:**	
Training of CMWs	2
Supervision of CMWs	2
Implementation of Program	1

**Table 2:** Demographic details of midwifery students

Midwifery students	*N* = 5
**Age**	
16–25	4
26–35	1
**Education**	
Intermediate	5
**Marital status**	
Unmarried	4
Married	1

**Table 3:** Demographic details of working and non-working group of midwives

Midwives	*N* = 10
**Age**	
21–25	2
26–30	1
31–35	4
36–40	3
**Education**	
Intermediate	5
BA	2
MA	3
**Midwifery course**	
12 months midwifery diploma	3
18 months midwifery diploma	7
**Workplace:**	
NGOs	4
Private organizations	5
Hospitals	1
**Working experience as midwife**	
Less than 5 years	2
Less than 10 years	5
More than 10 years	2
**Marital status**	
Unmarried	2
Married	8

**Table 4:** Demographic details of community women

Community women	*N* = 5
**Age**	
16–30	1
31–45	3
> 45	1
**Education**	
None	2
Primary	1
College	2
**Working status:**	
Currently working	3
Non-working	2

All the participants (*n* = 25) who were approached by the study team agreed to participate. Data was collected around two overarching themes of the CMW model (I) CMWs skills and competencies; and (II) ownership and supportive supervision. The themes and subthemes are presented in Table 5.

**Table 5:** Themes and subthemes

Themes	Subthemes
CMWs skills and competencies	• Pre-service training of CMWs • CMWs skills and competencies for service provision
Ownership and Supportive supervision	• Monitoring and supervision of CMWs • Ownership of CMW program • NO CMW regulatory body

### CMWs skills and competencies

#### Pre-service training of CMWs

Midwifery school, Thatta involves an admission committee intended to enroll midwifery students from the areas, where there are no established community clinics. The admission committee comprises of Principal of the midwifery school, the district coordinator for the MNCH program, and district officials (health) of district Thatta. The students have to undertake a written and an oral exam for their selection to the midwifery diploma program. The students get selected based on the need for birthing stations in specific areas of the district, and not on the basis of merit requirements.

*“Admission is done district-wise. We take a written exam and interviews for the new selection. Around 120 to 150 candidates appear for the exam but our criterion is according to seats of vacant posts. We select 30 students from Union Councils where there is a need for CMWs” (Principal- Midwifery school)*

*“Midwives are selected from those Union Councils where there is a need for CMW. There is an admission committee in which there is someone from the district office, a district coordinator, the principal of the midwifery school, and a few members from the MNCH office. This makes a committee of 6 to 7 people who do the selection” (District Coordinator MNCH)*

In their interviews, midwifery students acknowledged the learning opportunities they get during their classroom training and during practicums arranged at the midwifery school under the supervision of tutors.

*“We perform practicums in school*. *We learn on dummies about the stages of labor, about cervix dilation and its assessment, and also about the obstructed labor and its treatment” (Midwifery student)*

*“Our teachers work very hard. They really want us to save the lives of mothers and newborns in the future. They want us to be popular through the practice of our skills” (Midwifery student)*

According to the International confederation of midwives, global standards for competencies of midwifery educators include completion of a midwifery education program in theory and practice, registration and licensure to practice midwifery, and two years of completion of midwifery experience [17]. However, there are three tutors in the midwifery school of Thatta. None of the tutors has midwifery qualifications. There is one staff nurse and two doctors who teach midwifery courses. The training of midwifery students through teachers with no background in midwifery education and practice compromises their standard of training and clinical skills as stated by key informants.

*“Midwives can be trained only by teachers and principals who have a background in midwifery. We see our midwives being trained by doctors and nurses. They can only teach them theory-based learning. This is why we don’t have skilled midwives” (General secretary- Midwifery Association of Pakistan)*

For the practice of clinical training and to gain hands-on experience, midwifery students are assigned to a public sector District hospital, in Thatta, which is a prerequisite for completion of a midwifery diploma. Students are generally assigned to the obstetrics and gynecology department of the hospital to perform skills under the supervision of staff nurses. However, they usually assist nurses in basic vital signs monitoring and hygiene care of patients, as it was cited by midwifery students:

*“We go to civil hospital for our clinical rotations but we don’t perform the skills taught to us during school practicums, we mostly assist senior staff nurses in basic hygiene care and drug administration” (Midwifery student)*

Nearly all the students mentioned that they were not allowed to conduct deliveries and were not provided with any supervision in case they ever got an opportunity to conduct a normal vaginal delivery. This finding was supported by one of the key informants:

*“Students go to civil hospital and practice their skills but the staff present there does not cooperate. Some students get access to the wards and labor room but not all can practice” (District coordinator MNCH program)*

*“We never get the opportunity to perform normal deliveries during hospital rotations. Maybe this is because the hospital staff thinks that we can make mistakes and we are still not eligible to do these cases” (Midwifery student)*

Almost all key informants mentioned deficiencies in the clinical training of community midwifery students. They mentioned that midwifery students do not get exposure to community clinics or birthing stations during their clinical training. Moreover, they are not provided with opportunities to liaise with senior trained and deployed CMWs in the district. This lack of exposure to community clinics and to communities does not prepare students to work in communities as stated by one of the respondents:

*“The lack of exposure of community clinics to students is a problem. Students should get exposure to community clinics during their practice. They only work in hospitals and clinical settings during their training, then how can we expect them to work in an entirely different community setting. They are CMWs which means that they have to serve their communities” (General secretary- Midwifery Association of Pakistan)*

#### CMWs skills and competencies for service provision

Midwives develop various skills while providing services in clinics or communities over time. On their own, they learn counseling skills, deal with complicated cases, delivery practices, antenatal assessment, and various maternal and newborn services. Working midwives shared their experiences and learning opportunities they get during their work.

*“We deal with many high-risk patients with increased blood pressure and many other risks. But with working experience, we have learned to deal with difficult cases” (Working midwife)*

All the working CMWs who were interviewed expressed association of their success, with the development of trust with the communities. Some CMWs acknowledged using their professional skills to gain the trust of the communities. A midwife’s behavior and performance attract the community toward CMWs services.

*“…if training is good, if midwife’s behavior is good and the way they conduct deliveries is good, people prefer to go to midwives.” (Working midwife)*

Director General Nursing and Midwifery mentioned that CMWs with hands-on experience and a proficient set of skills could generate good revenue as they are licensed to run their own clinics independently without any supervision.

*“CMWs become self-sufficient when they start practicing their skills and they can earn a good amount if they have well-established clinics” (Director-general nursing and midwifery)*

However, no proper training of CMWs is a matter of concern as only the motivated ones are successful in communities and they learn by gaining experience over time, which could result in harm to the pregnant women, fetus, and/or a newborn. Proper monitoring of newly trained CMWs who are deployed in the community is required.

***“****Out of 100 girls trained, only 5 to 10 open their birthing stations in communities and there again they face a lot of challenges. Most of the time midwives are assigned to medical and surgical wards during their training. The true midwives we want to produce are very few in Pakistan. Otherwise, girls do training for the sake of diploma, the midwives we want who can go to fields with proper training to provide services in communities are very few”. (Director-General Nursing and Midwifery)*

### Ownership and Supportive supervision

#### Monitoring and supervision of CMWs

The monitoring and supervision of the CMW program are inefficient as responded by all key informants. Because of inefficient monitoring and supervision, CMWs services delivery remains ineffective. CMW program is one of the vertical programs of the MNCH program in the province of Sindh. The financial deficiencies of the MNCH program hinder the monitoring and supervision of CMWs of the district.

*“…the late payment to the district coordinator that coordinates and visits birthing stations is a problem. They require some expenditure to visit clinics. MNCH program is facing financial issues which affect monitoring of CMWs of the district” (District Health Officer)*

The lack of support and supervision from the MNCH program for example non-provision of stipend, lack of monitoring of CMWs, and non-support for establishing birthing stations in their communities resulted in non-coordination of CMWs with the district coordinator of the MNCH program. CMWs were required to provide monthly reports to the MNCH coordinator. However, out of 110 deployed community midwives in the district, only seventeen CMWs were providing monthly reports to the MNCH program coordinator. The argument provided by CMWs for non-coordination and non-provision of monthly reports exhibited non-supportive actions of the MNCH program.

*“We used to provide monthly reports to the program but we were not getting any incentive in return for it, so we have stopped reporting to them” (Working midwife) *

*“CMWs were supposed to report to us every month. They stopped providing us the reports because of the low-cost stipend that was provided after a long delay, they started taking the jobs and services offered by NGOs and they got disengaged with us (District coordinator- MNCH program)*

The monitoring and supervision of training schools are inadequate as reported by key informants. The lack of monitoring of midwifery schools affects the quality of CMWs being prepared at schools.

*“We don’t know the quality of CMWs we are producing because we have no monitoring and supervision system for training institutes” (Director- General nursing and midwifery)*

#### Ownership of CMW program

The majority of the study respondents reported about lack of ownership of the CMWs program at the national, provincial, and district levels. The lack of coordination to regulate and implement the program is a hindering factor for CMWs services. The MNCH program is directed by top-down management, where district managers are employed at the district level but are administered by the provincial government. The lack of coordination between the managers employed at different levels interposes the service structure of the CMWs program.

*“There is no ownership to monitor CMWs. The MNCH is responsible to plan and implement CMWs program but no one takes the ownership to monitor and fill the gaps and set targets. We do not have a defined structure. If we talk about MNCH, we don’t know about its responsibilities. MNCH looks after CMWs training, but who is responsible to monitor the indicators of MMR and IMR. We don’t have a definite answer”. (General secretary- Midwifery Association of Pakistan).*

The government of Pakistan set some incentives for midwifery students to facilitate their needs and requirements during training and for deployed CMWs to enable them in the attainment of their catchment populations. Most of the study respondents discussed non-provision and deferral of provision of incentives to midwifery students and deployed CMWs. The non-provision of incentives to midwives of the district discouraged them to work at their birthing stations and they acquired jobs where they could get a fixed salary.

“F*rom last 2 years our students are not getting their stipend. There is an issue of students stipend, we have complained about it multiple times, but they don’t do anything” (Principal- School of Midwifery)*

*“Government used to provide us PKR 2000 ($13) after training every month. Since many years, they have stopped this. Now CMWs find work in hospitals and BHUs to earn money” (Non-working midwife)*

Many efforts have been made by the MNCH program for midwives such as the provision of supplies, furniture, and equipment to establish birthing stations. Despite the efforts, CMWs are not effectively utilizing their services because of late payments to midwives, late payments to district coordinators, and the non-organization of proper service structure for CMWs deployment.

*“..The late payment to CMWs is a problem. They have to earn money. Another reason is the late payment to the district coordinator. They have to coordinate and visit birthing stations and for that, they require some expenditure. Payments are deferred for 1 to 2 years, so we do not go for visits, because we cannot pay the visit costs from our pockets. There is no proper service structure. If CMWs are trained we do not have service structure to appoint them to RHCs and BHUs” (District coordinator MNCH program)*

All the CMWs interviewed were employed with NGOs and private organizations. None of them were working at their birthing stations. They were working with organizations where they could earn a fixed amount of salary each month. As CMWs do not have a Basic Salary (BS) scale, every organization set CMWs’ salaries to their choice. They are not provided permanent jobs in the public health system of Pakistan, and the non-provision of government jobs for CMWs is the key hindering factor to the utilization of their services.

*“Government should have provided us government setups or jobs after our training. We are struggling in NGOs for the jobs. People are poor in villages. They do not pay for our services. We have to work in NGOs to earn money” (Working midwife)*

#### No CMWs regulatory body

Pakistan Nursing Council (PNC) is the regulatory body of CMWs. It provides curriculum and certification to CMWs. Thus far, the role of PNC for training and career development for CMWs is undefined and unclear. As the name Pakistan Nursing Council highlights Nursing in its name, it dominantly contributes to the development and growth of nurses as reported by one of the key informants:

*“We have 2 years midwifery program. PNC only provide us curriculum and very rarely and hardly any support is provided if there is any legal issue or any legal obligation then PNC provides support to CMWs” (Director General nursing and midwifery)*

CMWs of Pakistan face many challenges for career growth. There is a lack of higher education that has added to the indistinctness of the midwifery profession as cited by one of the key informants:

*“For nursing, we think a lot about higher education but for midwifery there is nothing. The midwives who are working will die in this profession being a midwife. There is no growth for them” (Director-general nursing and midwifery)*

## Discussion

The current maternal mortality ratio is 186/100000 live births, a reduction from 276/100000 live births from the 2006–7 survey [10, 11]. Even though maternal mortality has come down, the rate of reduction is low. CMW program is an important element of the MNCH program and it can improve skilled care in rural areas of Pakistan if implemented properly. The Government of Pakistan initiated the CMW program in 2006 [13]. Since then, many CMWs have been deployed in the district. However, the utilization of maternal and newborn services by CMWs is debatable.

The study has identified important factors that influence CMWs’ services such as their classroom training, hand-on skills, support for clinical practicums, monitoring of training institutions, support for job provision, and support for curriculum and fieldwork. We identified only a few facilitators under the domain of CMWs training yet there were no facilitating factors from the domain of supportive supervision. This marks that these findings are imperative to the program implementers and stakeholders involved in training and particularly in the monitoring and supervision of the CMW program.

The study findings suggest that the academic and clinical training of the CMW program has administrative weaknesses. Midwifery students were performing routine tasks during their clinical placements and not the skills that were taught to them in their school. The caveat in the training is the lack or almost absence of hands-on practice for conducting normal vaginal deliveries. Porter and colleagues [31] suggested that midwifery and nursing students have to be given opportunities to practice different tasks to become perfect and learn from their inaccuracies. However, the students were not able to practice skills in the clinical setting which could lead to a lack of success in requisite competencies and in turn compromise the care given to community women. Similar findings were reported by Australian nurses who reported dissatisfaction when they were not allowed to perform skills taught to them during their school practicums [32]. Teaching hospitals and midwifery training institutions should work together and plan strategies for clinical practicums. This strategy would not only enhance the learning of midwifery students but also increase the hospital workforce to provide patient care.

The results of the study suggest that the hospital staff does not support midwives during their clinical rotations. Literature suggests that midwifery educators or senior midwives are expected to accompany students to their clinical areas [33]. However, this might not be possible in Pakistan due to the lack of academic staff with midwifery training and the absence of senior midwives in public hospitals. Lack of clinical guidance and supervision has been identified as a major barrier by various studies where students learn incorrect procedures, become incompetent, and lose interest in clinical skills [33–37].

The study results also suggest that the training lacks on how to run a small business by establishing their own home clinic or a ‘birth station’ and marketing a small business. As CMWs are not employed by the Government and are expected to earn their income by charging fees for services, it is expected for them to establish birth clinics and operate business to support them financially. This major barrier was identified by numerous studies [38–40], and based on this need, a large-scale project by the Greenstar Social Marketing (NGO) and the faculty midwives from the Aga Khan University School of Nursing and Midwifery was organized aimed at providing practical training to midwives on business skills [41]. The training workshops were well received by participating CMWs and helped in satisfying a noticeable gap to establish birthing clinics within a business model [41].

Even though the study findings suggest that there is also a lack of ownership and administrative support for the CMW program by the government at the federal, provincial, and, district levels. The weak arrangement of the program at the district level has been highlighted as the main hindering factor for CMWs services. There is a lack of power to district-level managers and the district health system has inadequate administrative and financial control to implement the program. The provincial MNCH program is directed by top-down management where the roles of district officials are not properly defined and the program is financed by provincial authorities which is apparent by fragmentation in the program at the district level [42]. The financial limitations of the MNCH program have become a major threat to the CMW program’s sustainability as midwifery students and midwives are provided stipends after a long delay. These findings have been supported by MNCH program managers involved in the implementation of the CMWs program [43]. Due to the financial constraints of the MNCH program, almost all the vertical programs functioning under MNCH are disadvantaged [42].

The non-provision of jobs to CMWs in the public health system and no fixed Basic Salary scale for CMWs is an additional barrier to CMWs’ services. The lack of Government support for the provision of government jobs demotivates them to continue their work and they hunt for fixed jobs at different NGOs and private organizations other than the field, they were trained for. The non-retention of CMWs in their professions has been reported in almost all provinces of Pakistan [36, 37]. The major reason for CMWs’ non-retention from two provinces of Pakistan including Punjab and Khyber Pakhtunkhwa confirms similar findings of non-governmental support [43].

Findings of the study suggest that the regulatory body of CMWs ‘the Pakistan Nursing Council’ only provides curriculum and licenses to CMWs. Pakistan Nursing Council as the name suggests only possesses Nursing regulations and policies as its priority. Literature suggests that CMWs lack autonomy and self-sufficiency in countries where they do not have autonomous and independent regulatory bodies such as Nepal and Bangladesh [44].

Our study has several strengths. First, the exploratory design of this study helped us to understand the factors from all levels of the CMW program from organization to training and implementation. Second, the rigor of the study was achieved by enhancing the credibility of the findings from different data sources. Third, the study offers midwives’ own perspectives on their working conditions and their own thoughts about possible actions to improve them. This can create a basis for further interventional research that can be adapted to the local context. The study also has some limitations. First, the data collection was done through telephonic interviews because of the pandemic situation and the facial expressions and physical observations were therefore missed during the data collection. Secondly, the study focused only on the perspective of the participants who are direct or indirect part of the MNCH or CMW program.

In order to address the effective utilization of CMWs services, the study recommends intervening at all levels of the CMW program from planning to implementation. Firstly, the study provides insights to program implementers to rearrange the selection and deployment of CMWs and the training requires strengthening clinical hands-on practice. At least two days in teaching hospitals or primary care BHUs should be reserved for midwifery students to gain experience and get hands-on training. Midwifery students should be taught by certified and experienced midwives and the program should be adjusted to the local context in order to ensure sufficient exposure to community clinics and birthing stations. Moreover, the inclusion of the internship program as part of their training focused on strengthening clinical skills could be a positive addition to the program. Secondly, the MNCH program should work on strategies to empower CMWs and teach them to become respectable entrepreneurs rather than spoon-feeding them. The autonomy and authority of district-level managers for the implementation of the CMW program are very important to apply within the local context. Lastly, PNC should organize a separate division for regulations and curriculum development of CMWs and the inclusion of midwives in its title can gain recognition of this regulatory body working for CMWs.

## Conclusion

In conclusion, community midwives can play a vital role in reducing maternal and newborn mortality rates and in achieving progress in health service delivery. However, the findings suggest that these improvements cannot be made until the MNCH program and public health system takes action for re-organization and planning of the CMW program. The study provided an extensive understanding of factors influencing CMWs’ utilization of services in district Thatta.

This exploratory study provided us with reflections of midwifery students, administrative stakeholders of the MNCH program and district managers, CMWs working and not working in the district, and the community married women about the factors such as CMWs training, skill competencies, and the supportive supervision that influence community midwives utilization of services in district Thatta. It helped us to understand the factors from all levels of the program from organization to training and implementation of the CMWs program. The study has identified only a few facilitators for CMWs skills and competencies yet many serious gaps in the CMWs program at the level of clinical training, deployment, ownership, monitoring, and supportive supervision. These gaps are thought-provoking for policymakers and program implementers to reorganize the CMWs program.

In order to address the effective utilization of CMWs services, the study recommended intervening at all levels of the CMW program from planning to implementation. A well-trained, properly deployed, and supervised CMW can deliver the required services in rural areas of Pakistan. This can help to achieve the aim of introducing the CMW program in Pakistan. However, disregarding the supervision and training of CMWs in the health system, the aim of development of this cadre cannot be achieved.

## Supplementary Information

**Additional file 1. **COREQ (COnsolidated criteria for REporting Qualitative research) Checklist.

**Additional file 2. **Interview Guide.

**Additonal file 3. **Thematic Map.

## Data Availability

All data generated or analyzed during this current study available from the corresponding author on reasonable request.

## Abbreviations

ANC: Antenatal care
CMWs: Community Midwives
DHO: District Health Officer
EPI: Expanded Program for Immunization
FP: Family Planning
HCPs: Health Care Providers
IDI: In-Depth Interviews
KIIs: Key Informant Interviews
LHVs: Lady Health Visitors
LHWs: Lady Health Workers
LMICs: Low Middle-Income Countries
MAP: Midwifery Association of Pakistan
MMR: Maternal Mortality Rate
MNCH: Maternal, Newborn and Child Health
PNC: Pakistan Nursing Council
SDG: Sustainable Development Goal
TBAs: Traditional Birth Attendants
WHO: World Health Organization
