# Supplementary material for: Facilitators and barriers influencing utilization of services provided by community midwives in district Thatta, Pakistan: a qualitative exploratory study
Source: BMC Pregnancy Childbirth. 2022 Jun 22;22:506. doi: 10.1186/s12884-022-04823-8 (PMC9215135; doi:10.1186/s12884-022-04823-8)
Supplement: Supplementary file 2 — Additional file 2. Interview Guide. [file 12884_2022_4823_MOESM2_ESM.pdf]

Interview Guide

Key informant interview with District Health Officer

**CMWs skills competency**
1. What services are provided at primary facilities with maternal and newborn services and who provides the care?<br>*Probes: deliveries, perinatal care, home visits, immunization, growth monitoring provided by CMWs, LHVs*
2. Are midwives involved in the provision of services? And at what level of care?<br>*Probes: deliveries, perinatal care, home visits, immunization, growth monitoring provided by CMWs, LHVs, levels of care*
**Supportive supervision**
3. What are the factors that facilitate midwifery services in the district? Does your office provide any support to midwives to practice in the district?<br>*Probes: Cultural context, social context, transport, language*
4. In your view what are important barriers to maternal and newborn health services in the district?<br>*Probes: lack of information, inadequate staffing, sociocultural barriers*, c*ultural context, social context, transport, language*
5. What is the process of training and deployment of midwives in the district? Does your office have any role in deploying CMWS after the training is completed?<br>*Probes: how midwives are trained, who deploy, how many leaders are involved in the process?*

6. What support is provided to midwives from government sector?<br><br>*Probes: supervision, training, deployment*

Key informant interview with Principal, School of midwifery

**CMWs skills competency**
1. What courses are covered in midwifery education?<br><br>*Probes: practical and theory work, courses and curriculum*
2. Where and how midwives are trained for clinical practice? Are these opportunities sufficient to start independent practice?<br><br>*Probes: supervised deliveries, newborn assessments, antenatal checkups*
3. What is the educational background of midwifery teachers?<br><br>*Probes: midwives, nurses, geographical location, practical work*
4. What assessment criterion is followed to provide certification?<br><br>*Probes: theory exams, clinical exams, grading criteria*
5. What measures are followed for students who do not successfully pass their exams?<br><br>*Probes: re-sit exams, repetition of annual term*
6. Do you have any appreciation reward for high scoring students? If yes, what is it?<br><br>*Probes: financial rewards, certificates, material rewards, jobs allotment*
**Supportive supervision**
7. What is the admission criteria and process of enrollment for midwifery certification?<br><br>*Probes: admissions, qualification, enrollment forms, quota system, admission test*

8. In your opinion, time required for training midwives is appropriate taking in consideration theory and practical knowledge?<br>*Probes: theory knowledge and practicums*
9. What is the involvement of stakeholders in training of midwives and who do you report?<br>*Probes: reporting to stakeholders at district level and national level*
10. What is the fee structure for midwifery certification?<br>*Probes: fee charged, semesters and term system, annual system*

Key informant interview with President, Midwifery Association of Pakistan

**Supportive supervision**
1. What are the major health issues related to maternal and newborn health in Pakistan?<br>*Probes: maternal and newborn mortality, access to antenatal and delivery services, postpartum complication, complication related to abortions and deliveries*
2. What is the objective of Midwifery Association of Pakistan?<br>*Probes: career development, trainings, quality of curriculum*
3. What support Pakistan Nursing Council and Government provide to midwives in trainings, deployment, building linkages with communities, other health care providers, for referrals?<br>*Probes: financial incentives, trainings, curriculum, career development*
4. In your opinion, what motivates midwives to choose midwifery as their profession?<br>*Probes: money, interaction with women as their clients, social and cultural preferences*
5. What are the challenges to midwives for delivery of services?

Probes: finance, community support, social and cultural barriers, respect of job, dignity of profession
**CMWs skills competency**
6. What is the importance of CMWs in provision of maternal and newborn services?<br>*Probes: maternal and newborn services, easy community access, home visits*
**7.** Are midwives involved in the provision of maternal and newborn services? And at what level of care?<br>*Probes: deliveries, perinatal care, home visits, immunization, growth monitoring provided by CMWs, LHVs, levels of care*
8. What is the quality of pre-service training of midwives?<br>*Probes: hands-on clinical practice, clinical and public health knowledge*

Key informant interview with Coordinator, Maternal and Neonatal Child Health Program

**CMWs skills competency**
1. What services are provided at primary facilities with maternal and newborn services and who provides the care?<br>*Probes: deliveries, perinatal care, home visits, immunization, growth monitoring provided by CMWs, LHVs*
2. Are midwives involved in the provision of services? And at what level of care?<br>*Probes: deliveries, perinatal care, home visits, immunization, growth monitoring provided by CMWs, LHVs, levels of care*

**Supportive supervision**
3. What are the factors that facilitate midwifery services in the district? Does your office provide any support to midwives to practice?<br>*Probes: Cultural context, social context, transport, language*
4. In your view what are important barriers to maternal and newborn health services?<br>*Probes: lack of information, inadequate staffing, sociocultural barriers*, *cultural context, social context, transport, language*
5. What is the process of training and deployment of midwives? Does your office have any role in deploying CMWS after the training is completed?<br>*Probes: how midwives are trained, who deploy, how many leaders are involved in the process?*
6. What support is provided to midwives from government sector?<br>*Probes: supervision, training, deployment*

Key informant interview with Director, General Nursing and Midwifery

**Supportive supervision**
9. What are the major health issues related to maternal and newborn health in Pakistan?<br>*Probes: maternal and newborn mortality, access to antenatal and delivery services, postpartum complication, complication related to abortions and deliveries*
10. What support Pakistan Nursing Council and Government provide to midwives in trainings, deployment, building linkages with communities, other health care providers, for referrals?

Probes: financial incentives, trainings, curriculum, career development
11. In your opinion, what motivates midwives to choose midwifery as their profession?<br>*Probes: money, interaction with women as their clients, social and cultural preferences*
12. What are the challenges to midwives for delivery of services?<br>*Probes: finance, community support, social and cultural barriers, respect of job, dignity of profession*
**CMWs skills competency**
13. What is the importance of CMWs in provision of maternal and newborn services?<br>*Probes: maternal and newborn services, easy community access, home visits*
**14.** Are midwives involved in the provision of maternal and newborn services? And at what level of care?<br>*Probes: deliveries, perinatal care, home visits, immunization, growth monitoring provided by CMWs, LHVs, levels of care*
15. What is the quality of pre-service training of midwives?<br>*Probes: hands-on clinical practice, clinical and public health knowledge*

In-depth Interviews with midwifery school students

**Demographic data**		
1.	Age	

2.	Qualification	
3.	Marital status	

**General questions**
1. Why do you want to become a midwife?<br><br>*Probes: self-choice, only option, parental pressure*
2. What are your views about significance of midwifery profession?<br><br>*Probes: views about scope, earning, respect and dignity*
3. What advice would you give to people considering midwifery as a career?<br><br>*Probes: preferences about midwifery as a profession and why?*
**CMWs skills competency**
4. How do you find curriculum and training of your program?<br><br>*Probes: clinical and practical work, theory knowledge, hands on practice, quality of teachers*
5. What services can you provide after training?<br><br>*Probes: knowledge about services of midwives*
6. What opportunities are provided to you to experience midwifery skills?<br><br>*Probes: competence to practice skills, confidence to perform deliveries, perinatal assessments*
7. What are your views about course plans and assessment criteria?<br><br>*Probes: theory and practical together, assessments related to theory and practical work*
8. Where will you work after your training is completed?<br><br>*Probes: community centers, hospitals, maternity centers*
**Supportive supervision**

9. How do you find support to practice clinical skills?<br><br>*Probes: support of instructors, support of community, support of in-service midwives*
10. In your opinion what are some reasons that lead midwives to discontinue their profession? What other jobs they are seeking for?<br><br>*Probes: social and cultural barriers, competence, money, community support, stakeholders support*
11. What are facilitating factors for CMWs who have well-established setups in district communities?<br><br>*Probes: community trust, competency of skills, stakeholders support, money*

In depth Interviews– working group

**Demographic data**		
1.	Age	
2.	Type of midwifery qualification	
3.	Highest education level	
4.	Year of qualification	
5.	Employment status	
6.	Years of experience	

7.	Marital status	

**CMWs skills competency**
1. What is your current role in the facility?<br><br>*Probes: services, teamwork, duties*
2. What is your opinion about your pre-service midwifery training for provision of maternal and newborn services?<br><br>*Probes: knowledge about maternal and newborn services, competence of clinical practice*
3. What services do you provide to community women without supervision?<br><br>*Probes: counseling, education, deliveries, antenatal care, detection of high risk cases, newborn assessments, referral services*
**Supportive supervision**
4. What support is provided by government to you?<br><br>*Probes: supervision, follow-up of the services, finance, setup of clinics*
5. For how long have you been working as CMWs?<br><br>*Probes: money, community approach, support from government and private sector, respect*
6. What are the challenges you have faced? What were the facilitator factors which helped you in continuing your work as CMW?<br><br>*Probes: money, community approach, support from government and private sector, respect*
7. Have you considered leaving your job? If so, why? What factors contributed to your decision to leave the job?<br><br>*Probes: security, gender issues, personal issues, social and cultural issues*

8. What advice would you give to people considering midwifery as a career?<br>*Probes: preferences about midwifery as a profession and why?*
9. What are your views about midwives who are not performing their role for maternal and newborn services?<br>*Probes: involved in other activities, social, cultural and workplace issues*
10. What are your opinions about availability and accessibility of midwives for maternal and newborn services in your district?<br>*Probes: role of midwives, midwives for maternal and newborn services*

In depth Interviews - Non working group

**Supportive supervision**
1. What support is provided by government and stakeholders to midwives of your district?<br>*Probes: supervision, follow-up of the services, finance, setup of clinics*
2. What are the challenges to midwives for delivery of services?<br>*Probes: finance, community support, social and cultural barriers, respect of job, dignity of profession*
3. What support was provided to you when you were in your job?<br>*Probes: finance, community support, support by stakeholders*
**CMWs skills competency**
11. What is your opinion about your pre-service midwifery training for provision of maternal and newborn services?<br>*Probes: knowledge about maternal and newborn services, competence of clinical practice*

IDI with community women

Demographic data		
1.	Age	
2.	Education	
3.	Marital status	
4.	Number of children	
5.	Available nearby facilities	

**Supportive supervision**
1. What are your views about accessibility for midwifery services?<br><br>*Probes: transport, time, location*
**CMWs skills competency**
2. What are your views about knowledge of midwives for maternal health?<br><br>*Probes: knowledge about high risk pregnancies, complications, newborn care*
