# Supplementary material for: Facilitators and barriers influencing utilization of services provided by community midwives in district Thatta, Pakistan: a qualitative exploratory study
Source: BMC Pregnancy Childbirth. 2022 Jun 22;22:506. doi: 10.1186/s12884-022-04823-8 (PMC9215135; doi:10.1186/s12884-022-04823-8)
Supplement: Supplementary file 3 — Additonal file 3. Thematic Map. [file 12884_2022_4823_MOESM3_ESM.pdf]

# THEMATIC MAP

| Condensed meaning units | Codes | Subthemes | Themes |
|---|---|---|---|
| Curriculum about maternal and child health | **Learning opportunities during classroom trainings and practice of their skills** | | |
| Practical learning at hospital | | | |
| Teachers and staff help in learning skills Going civil hospital during training | | | |
| Practicum in school and practice in hospital | | | |
| Learn from assessments | | | |
| Senior midwives and teachers support in learning | | | |
| Learn on dummies | | | |
| Shifting duties in civil hospital during training | | | |
| Skills of delivery, ANC and PNC learnt during training | | | |
| Maternal and child health courses and knowledge about vaccination | | | |
| Clinicals in maternity and newborn wards | | | |
| Classroom and clinical training | | | |
| Students learning opportunities | | | |
| Time period and process of training | | | |

| | | | |
|---|---|---|---|
| Learning from clinicals | | **Pre-service training of CMWs** | |
| Practice on dummies | | | |
| Learning in skills lab | | | |
| Learning on dummies | | | |
| Midwives skills development | | | |
| Learning from clinicals | | | |
| Tutors support | | | |
| Lack of competency of tutors | | | |
| Educational background of teachers | **Educational background of midwifery tutors** | | |
| Educational background of teachers | | | |
| Midwives taught by doctors and nurses | | | |
| No background information on midwifery skills | | | |
| No midwife available to teach midwives | | | |
| Admissions according to union councils | **Admission procedure of CMWs** | | |
| Preadmission exam details | | | |
| Age criteria for admission | | | |
| Selection of girls from UCs of low education rate | | | |
| Admission done by admission committee | | | |
| Admission committee consists of 5 to 6 people | | | |
| Admission test for CMWs | | | |
| Girls selected from those UCs where no midwife available | | | |

| | | | |
|---|---|---|---|
| Less midwives enrollment from deprived UCs | | | **CMWS SKILLS AND COMPETENCIES** |
| No knowledge about referral to students | **Deficiencies in midwifery training** | | |
| Learn about maternal and child health during training | | | |
| Skills of IM injection and drug dosage during training in hospital | | | |
| Perform IV and drug dosage during training in hospitals | | | |
| Learn skills of PV exam, IV administration, vital signs in hospital during training | | | |
| No visits to birthing stations | | | |
| Involvement of hospital administration | | | |
| Lack of competency building training | | | |
| Non-support of hospital staff in training students | | | |
| Competency from working in fields | | | |
| Competency of services with experience | | | |
| Sharing her experience of dealing with a complicated case | | | |
| Midwives are competent | | | |

| | | | |
|---|---|---|---|
| Competency to conduct deliveries, ANC, PNC with time and experience | **Development of skills with working experience** | **CMWs skills and competencies for service provision** | |
| Challenge of dealing with high risk patients | | | |
| Competent to do deliveries | | | |
| Sharing her experience of IUD | | | |
| Midwives to perform Antenatal checkups to prevent complications | | | |
| Community prefer midwives because of her behavior | **Professional skills to gain trust of the communities** | | |
| Earning based on skills | | | |
| Time and attention of CMWs to gain community trust | | | |
| Midwives provide privacy to patients | | | |
| Midwives listen to community concerns | | | |
| CMWs socially and culturally acceptable care | | | |
| CMWs know people of their communities and provide care accordingly | | | |
| CMWs provides time to clients to listen to their concerns | | | |
| CMWs are easily approachable | | | |

| | | | |
|---|---|---|---|
| CMWs behavior is a facilitator | | | |
| CMWs are licensed to open their clinics | | | |
| LHWs work under CMWs because | | | |
| CMWs is the only cadre after doctors to open their clinics | | | |
| The purpose of CMWs was to setup birthing stations | | | |
| They can provide independent care | | | |
| Provision of care without supervision | | | |
| Can perform ANC, PNC, FP and deliveries independently | **CMWs can provide independent care without supervision** | | |
| Only cadre to provide independent care in communities | | | |
| Left because of money | | | |
| Delay in stipend | | | |
| Delay in stipend provision | | | |
| No monitoring and supervision by MNCH coordinator | | | |
| Delay in provision of stipend to midwives | | | |
| Increase absenteeism due to delay in stipend | **Lack of monitoring and supervision and financial deficiencies of MNCH program** | | |
| No support in remote areas to perform deliveries | | **Monitoring and supervision of CMWs** | |
| MNCHs program funding problem | | | **OWNERSHIP AND SUPPORTIVE SUPERVISION** |
| Late payment to CMWs | | | |
| No service structure for CMWs to provide them jobs | | | |
| Decrease in coordinator and reporting to MNCH department | | | |

| | | | |
|---|---|---|---|
| Accelerated action plan for performance-only 17 out of 110 | | | |
| Late payments to MNCH coordinator | | | |
| Funding problems of MNCH program of Sindh | | | |
| No support for establishing birthing stations | | | |
| Problem of government incentive<br>Non provision of equipment by government | | | |
| Lack of monitoring of schools by PNC and government | | | |
| No monitoring and supervision of students during clinicals | | | |
| No stipend by government | **Lack of Government support** | **Ownership of CMW program** | |
| NO BS scale for CMWs | | | |
| CMWs are not recognized in government national program for health | | | |
| CMWs have no professional and educational growth | | | |
| Check and balance and action | | | |
| Continuation of failure | | | |
| Political involvement | | | |
| Lack of System structure | | | |
| Lack of stakeholders support from department of health | | | |
| No government jobs | | | |
| Poverty in villages | | | |
| PNC developed curriculum in 1973 | | | |
| PNC only provide support of curriculum and sometime of legal matters | | | |
| The time duration had also been changing | | | |

| | | | |
|---|---|---|---|
| The midwifery cadre had been varying over time | **Role of PNC in CMWs regulation** | **No CMWs regulatory body** | |
| CMWs are registered with PNC to provide care | | | |
| PNC accredited midwifery schools | | | |
| PNC provide midwifery curriculum and take exams | | | |
| PNC provides licensing to midwives | | | |
| No separate regulatory body for CMWs | **Lack of dominant role for CMWs** | | |
| No monitoring and supervision of CMWs | | | |
| Only provides curriculum | | | |
| No extra classes for CMWs | | | |
| No refresher trainings by PNC | | | |
| PNC doesn’t approach working midwives | | | |
| PNC looks after all nurses and midwives | | | |
| No domain for midwives | | | |
| No separate department for CMWs | | | |
